# MSH2, MSH6, MLH1, and PMS2 immunohistochemistry as highly sensitive screening method for DNA mismatch repair deficiency syndromes in pediatric high-grade glioma

**DOI:** 10.1007/s00401-025-02846-x

**Published:** 2025-02-02

**Authors:** Lea L. Friker, Thomas Perwein, Andreas Waha, Evelyn Dörner, Rebecca Klein, Mirjam Blattner-Johnson, Julian P. Layer, Dominik Sturm, Gunther Nussbaumer, Robert Kwiecien, Isabel Spier, Stefan Aretz, Kornelius Kerl, Ulrike Hennewig, Marius Rohde, Axel Karow, Ingmar Bluemcke, Ann Kristin Schmitz, Harald Reinhard, Pablo Hernáiz Driever, Susanne Wendt, Annette Weiser, Ana S. Guerreiro Stücklin, Nicolas U. Gerber, André O. von Bueren, Claudia Khurana, Norbert Jorch, Maria Wiese, Christian P. Kratz, Matthias Eyrich, Michael Karremann, Ulrich Herrlinger, Michael Hölzel, David T. W. Jones, Marion Hoffmann, Torsten Pietsch, Gerrit H. Gielen, Christof M. Kramm

**Affiliations:** 1 Institute of Neuropathology, DGNN Brain Tumor Reference Center, University Hospital Bonn, Venusberg-Campus 1, 53127 Bonn, Germany; 2 Institute of Experimental Oncology, University Hospital Bonn, Bonn, Germany; 3 Division of Pediatric Hematology and Oncology, Department of Pediatrics and Adolescent Medicine, Medical University of Graz, Graz, Austria; 4 Styrian Children’s Cancer Research, Research Unit for Cancer and Inborn Errors of the Blood and Immunity in Children, Medical University of Graz, Graz, Austria; 5 Hopp Children’s Cancer Center Heidelberg (KiTZ), Heidelberg, Germany; 6 Division of Pediatric Glioma Research, German Cancer Research Center (DKFZ) and German Cancer Consortium (DKTK), Heidelberg, Germany; 7 Department of Radiation Oncology, University Hospital Bonn, Bonn, Germany; 8 Department of Pediatric Oncology, Hematology and Immunology, Heidelberg University Hospital, Heidelberg, Germany; 9 Institute of Biostatistics and Clinical Research, University of Münster, Münster, Germany; 10 Institute of Human Genetics, Medical Faculty, University of Bonn, Bonn, Germany; 11 National Center for Hereditary Tumor Syndromes, University Hospital Bonn, Bonn, Germany; 12 Department of Pediatric Hematology and Oncology, University Children’s Hospital Münster, Münster, Germany; 13 Department of Pediatric Hematology and Oncology, University Hospital Giessen and Marburg, Giessen, Germany; 14 Department of Pediatrics and Adolescent Medicine, University Hospital Erlangen, Erlangen, Germany; 15 Comprehensive Cancer Center Erlangen, Erlangen, Germany; 16 Institute of Neuropathology, University Hospital Erlangen, Erlangen, Germany; 17 Department of Pediatrics, Asklepios Kinderklinik Sankt Augustin, Sankt Augustin, Germany; 18 Department of Pediatric Oncology and Hematology, Charité-Universitätsmedizin Berlin, Corporate Member of Freie Universität Berlin and Humboldt-Universität Zu Berlin, German HIT-LOGGIC-Registry for pLGG in Children and Adolescents, Berlin, Germany; 19 Department of Pediatric Oncology and Hematology, University Hospital Leipzig, Leipzig, Germany; 20 Department of Oncology, University Children’s Hospital Zurich, Zurich, Switzerland; 21 Children’s Research Center, University Children’s Hospital Zurich, Zurich, Switzerland; 22 Department of Pediatrics, Gynecology and Obstetrics, Division of Pediatric Hematology and Oncology, Geneva University Hospital, Geneva, Switzerland; 23 Department of Pediatrics, Gynecology and Obstetrics, CANSEARCH Research Laboratory, Faculty of Medicine, University of Geneva, Geneva, Switzerland; 24 Department of Pediatric Hematology and Oncology, Children’s Center Bethel, University Hospital Ostwestfalen-Lippe, Bielefeld, Germany; 25 Division of Pediatric Hematology and Oncology, University Medical Center Göttingen, Göttingen, Germany; 26 Department of Pediatric Hematology and Oncology, Hannover Medical School, Hannover, Germany; 27 University Children’s Hospital, University Hospital Würzburg, Würzburg, Germany; 28 Department of Pediatric and Adolescent Medicine and Mannheim Cancer Center (MCC), University Medical Center Mannheim, Medical Faculty Mannheim, Heidelberg University, Mannheim, Germany; 29 Department of Neurooncology, Center for Neurology and CIO ABCD, University Hospital Bonn, Bonn, Germany

**Keywords:** Pediatric high-grade glioma, Lynch syndrome, Constitutional mismatch repair deficiency, Immunohistochemistry

## Abstract

Pediatric high-grade glioma (pedHGG) can occur as first manifestation of cancer predisposition syndromes resulting from pathogenic germline variants in the DNA mismatch repair (MMR) genes *MSH2*, *MSH6*, *MLH1*, and *PMS2*. The aim of this study was to establish a generalized screening for Lynch syndrome and constitutional MMR deficiency (CMMRD) in pedHGG patients, as the detection of MMR deficiencies (MMRD) may enable the upfront therapeutic use of checkpoint inhibitors and identification of variant carriers in the patients’ families. We prospectively enrolled 155 centrally reviewed primary pedHGG patients for MMR-immunohistochemistry (IHC) as part of the HIT-HGG-2013 trial protocol. MMR-IHC results were subsequently compared to independently collected germline sequencing data (whole exome sequencing or pan-cancer DNA panel next-generation sequencing) available in the HIT-HGG-2013, INFORM, and MNP2.0 trials. MMR-IHC could be successfully performed in 127/155 tumor tissues. The screening identified all present cases with Lynch syndrome or CMMRD (5.5%). In addition, MMR-IHC also detected cases with exclusive somatic MMR gene alterations (2.3%), including *MSH2* hypermethylation as an alternative epigenetic silencing mechanism. Most of the identified pedHGG MMRD patients had no family history of MMRD, and thus, they represented index patients in their families. Cases with regular protein expression in MMR-IHC never showed evidence for MMRD in DNA sequencing. In conclusion, MMR-IHC presents a cost-effective, relatively widely available, and fast screening method for germline MMRD in pedHGG with high sensitivity (100%) and specificity (96%). Given the relatively high prevalence of previously undetected MMRD cases among pedHGG patients, we strongly recommend incorporating MMR-IHC into routine diagnostics.

**Supplementary Information:**

The online version contains supplementary material available at 10.1007/s00401-025-02846-x.

## Introduction

Central nervous system (CNS) tumors represent the second most frequent cancer in childhood and adolescence [34]. They can occur as first manifestations of cancer predisposition syndromes (CPS) [20, 21]. Data from pan-cancer studies using large-scale sequencing suggest that approximately 8% of all children with cancer carry an unambiguous cancer predisposing germline variant [15, 47]. With regard to aggressive CNS tumors in children, pediatric high-grade gliomas (pedHGG) occur most frequently in association with Li-Fraumeni syndrome [24]. Less common is the association of pedHGG with DNA mismatch repair deficiencies (MMRD). Although diffuse IDH-mutant astrocytomas mostly occur in adults and carry a more favorable prognosis than their wildtype counterparts [20], Suwala et al. described an epigenetic group of primary mismatch repair (MMR)-deficient IDH-mutant astrocytomas (PMMRDIA) found predominantly in children, adolescents, and young adults exhibiting a very poor overall survival (OS) similar to CNS WHO grade 4 IDH-wildtype gliomas [20, 41].

MMR proteins detect and excise nucleotide mismatches that spontaneously occur during DNA synthesis. Impairment of one of the MMR genes *MSH2*, *MSH6*, *MLH1,* and *PMS2* by pathogenic variants results in defective DNA repair mechanisms with subsequent oncogenic hypermutability and microsatellite instability (MSI) [20, 21]. Germline (constitutional) pathogenic variants in the MMR genes cause the autosomal dominant Lynch syndrome (LS), one of the most frequent CPS, and the rare autosomal recessive constitutional mismatch repair deficiency (CMMRD) syndrome. Both disorders are characterized by a higher incidence of tumors from the Lynch spectrum, including gastrointestinal, urogenital, hepatobiliary, skin, and brain cancer [46]. In a recently published study reporting 201 cases of children with CMMRD registered in the International Replication Repair Deficiency Consortium (IRRDC) across more than 50 countries, CNS tumors were the most frequent malignancies to occur (51%) [12]. Nonetheless, there is no routine screening for MMRD established for pedHGG. The diagnosis of LS or CMMRD is of high relevance for the initiation of preventive measures in the patients and variant carriers among their relatives. Furthermore, early detection of MMRD allows for individualized therapeutic decision-making.

Methods commonly used to screen for MMRD in adult solid Lynch spectrum tumors, in particular colorectal and endometrial cancers, are either testing for MSI or immunohistochemistry (IHC) for MMR protein expression [6, 8, 13, 23, 35, 37, 38, 45]. Compared to MSI testing, IHC using antibodies against the four MMR proteins MSH2, MSH6, MLH1, and PMS2 (referred to as MMR-IHC) represents a relatively widely available, affordable, and easy to perform technique [23, 37, 45]. MMRD is defined by a single or combinational loss of expression of MMR proteins, which is observed when genetic alterations affect transcription. So far, only single cases or small case series of pedHGG underwent immunohistochemical testing for MMRD [1, 14]. We therefore aimed to evaluate MMR-IHC as a screening approach for MMRD in a prospective cohort of 155 primary pedHGG cases.

## Materials and methods

### Patient selection

All patients ≤ 18 years of age with primary diagnosis of pedHGG undergoing central neuropathological review as potential candidates for the HIT-HGG-2013 (NCT03243461) trial at the Brain Tumor Reference Center of the German Society for Neuropathology and Neuroanatomy (DGNN) in Bonn, Germany, between August 2018 and October 2022 were included.

### Immunohistochemistry (IHC)

Formalin-fixed paraffin embedded (FFPE) tissues were cut into 4 μm-thick slides. Besides standard H&E staining, IHC to study the expression of DNA MMR proteins were performed using the monoclonal mouse anti-MSH2 (1:200, clone G219-1129, Cell Marque™, Rocklin, CA, USA), monoclonal mouse anti-MSH6 (1:50, clone 44, Cell Marque™), monoclonal mouse anti-MLH1 (1:20, clone G168-728, Bio SB, Goleta, CA, USA), and monoclonal mouse anti-PMS2 (1:50, clone MRQ-28, Cell Marque™) antibodies. Images were acquired using the Aperio GT 450 DX slide scanner (Leica Biosystems, Wetzlar, Germany) and Aperio ImageScope software (Leica Biosystems). For antibody testing and IHC protocol optimization, a control group was generated including tumor samples of nine patients with initial pedHGG diagnosis outside the screening period and confirmed germline MMRD.

### DNA panel next-generation sequencing

The TruSight Oncology 500 assay (TSO 500) was performed according to the manufacturer’s protocol [33]. The significance of detected variants was determined using the databases SIFT, PolyPhen-2, and ClinVar. Only variants with likely pathogenic to pathogenic significance were considered for analysis. 130 homopolymer MSI marker loci were analyzed to determine the MSI status.

### Polymerase chain reaction (PCR) and pyrosequencing of *MSH2*

To assess hypermethylation of *MSH2*, genomic DNA was extracted from FFPE tumor tissue and leucocytes and subsequently converted into bisulfite-DNA using the EpiTect® Bisulfite Kit (Qiagen, Hilden, Germany) according to manufacturer’s instructions. Primer for amplification of bisulfite-treated DNA were designed as follows: MLH2-f: 5′-gagtaaatatagaaaggagttt-3′ and MLH2-r1: 3′-aaacctcctcacctcctaatta-5′. The reverse primer MLH2-r1 was biotinylated at the 5’-end. The amplified region corresponds to chromosome 2, nucleotides 47,402,918 to 47,403,165. PCR was performed as previously described by Mikeska et al. [28]. The PCR product was subsequently subjected to pyrosequencing performed on the PyroMark® Q24 instrument (Biotage, Uppsala, Sweden) using PyroMark® Gold Reagents (Qiagen), according to manufacturer’s instructions and analyzed using PyroMark® CpG quantification software. The primer used for extension reaction was MLH2-p1: 5′-tagtagttaaagttattag-3′ (chromosome 2, 47,403,077 to 47,403,165). By calculating the mean of the arithmetic point of the first eight CpG dinucleotide positions, the overall methylation level was determined. Methylated converted DNA from EpiTect® PCR Control DNA Set (Qiagen) was used as positive control. DNA of a control patient with unmethylated *MSH2* was used as negative control.

### INFORM trial dataset

In the INFORM trial, detailed analysis of potential therapeutic targets was performed using whole exome sequencing (WES) of DNA extracted from both fresh frozen tumor tissue and ethylenediaminetetraacetic acid blood [44]. In addition, mRNA sequencing was performed in cases with available material. Access to the INFORM register was approved by the INFORM coordinators from Germany and Switzerland.

### Survival analyses

A reference cohort was generated from the HIT-HGG database of the (German-speaking) Society of Pediatric Oncology and Hematology GPOH including participants with the same diagnoses as the MMRD patients aged ≥ 3 to < 18 years prospectively enrolled in the HIT-HGG-2007 (ISRCTN19852453) and HIT-HGG-2013 (NCT03243461) trials (n[2007] = 38; n[2013] = 23). Reclassification of tumor diagnoses according to the fifth edition of the WHO Classification of CNS Tumors (WHO 2021) [20] was performed by an expert panel of neuropathologists and molecular biologists (L.L.F., G.H.G., D.S., D.T.W.J.). Patients with radiation-induced or secondary HGG and/or any known CPS were excluded from the reference cohort.

Survival analyses were performed using SPSS (version 29.0, SPSS Inc., Chicago, IL, USA). Inferential statistics were intended to be exploratory (hypotheses generating), not confirmatory, and were interpreted accordingly. The comparison-wise type-I error rate was controlled instead of the experiment-wise error rate. The local significance level was set to 0.05. No adjustment for multiple testing was performed. For univariate/multivariate survival analyses, log-rank test/Cox regression was applied, respectively. OS was calculated from date of initial diagnosis until death of any cause. Surviving patients were censored at the date of last follow-up. Event-free survival (EFS) was defined as date of initial diagnosis until occurrence of relapse or progression (date of histological confirmation or date of first sequentially confirmed MRI assessment), second malignancy, or death of any cause. Post-progression survival (PPS) represents the survival time between any of the events defined above (except death) and the date of death.

### Non-survival-related statistical analyses and data visualization

Data analysis was performed using GraphPad Prism 10 (GraphPad Software, San Diego, CA, USA) and Microsoft® Excel Version 16.86. Figures were generated using GraphPad Prism 10, Adobe Illustrator 2024 (Adobe Inc., Mountain View, CA, USA), and SankeyMATIC.com.

## Results

### Patient characteristics and neuropathological review

**Fig. 1:**
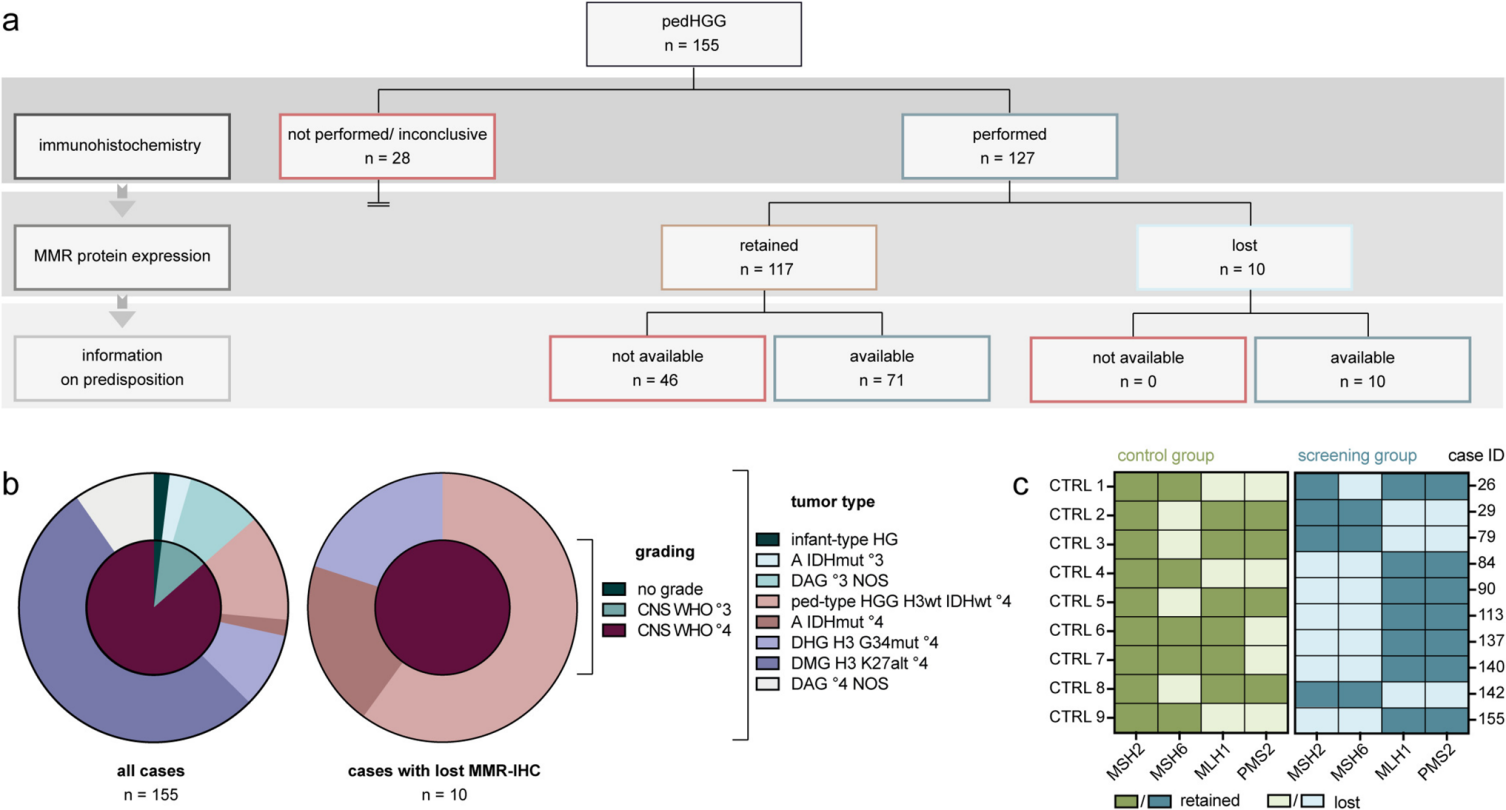
Study design and composition of the cohort. **a** Flowchart of study design. **b** Composition of the cohort displayed by grading (inner circle) and tumor type (outer circle) of all included patients (n = 155; left) and all patients with loss of MMR protein expression in MMR-IHC (n = 10; right). **c** Heat map of MMR-IHC results of control cases (left; green) and all cases suspicious of MMRD in the screening group (right; blue). The control group includes tumor samples of nine patients with initial pedHGG diagnosis outside the screening period and confirmed germline MMRD. This group was assembled for antibody testing and IHC protocol optimization. Case IDs are displayed on the y-axis. *A IDHmut °3/4* astrocytoma, IDH-mutant (CNS WHO grade 3/4), *CTRL* control, *DAG °3/4* diffuse astrocytic glioma (CNS WHO grade 3/4), *DHG H3 G34mut °4* diffuse hemispheric glioma, H3 G34-mutant (CNS WHO grade 4), *DMG H3 K27alt °4* diffuse midline glioma, H3 K27-altered (CNS WHO grade 4), *MMR(D)* mismatch repair (deficiency), *IDH* isocitrate dehydrogenase, *IHC* immunohistochemistry, *infant-type HG* infant-type hemispheric glioma, *NOS* not otherwise specified, *pedHGG* pediatric high-grade glioma, *ped-type HGG H3wt IDHwt °4* pediatric-type high-grade glioma H3-wildtype and IDH-wildtype (CNS WHO grade 4)

Following the HIT-HGG-2013 study protocol, tumor tissue samples of all 155 included patients with primary pedHGG were characterized by detailed histo-molecular analysis including MMR-IHC, depending on the amount of tissue available (Fig. 1a). The median age at diagnosis was 10.0 years (range 0.3–18 years) with a male predominance [92/155 (59%) male, 61/155 (39%) female, 2/155 (1.2%) n.a.]. 107/155 (69%) cases were initially classified according to the 2016 WHO classification of CNS tumors valid at the time of diagnosis and reclassified according to WHO 2021 for the purpose of this study (Fig. S1). The cohort was composed of 18 patients with CNS WHO grade 3 (12%), 134 patients with CNS WHO grade 4 glioma (86%), and 3 (2%) patients with gliomas without grading. The most frequent diagnosis was diffuse midline glioma, H3 K27-altered (CNS WHO grade 4) (DMG) (82/155; 53%), followed by diffuse pediatric-type HGG, H3-wildtype and IDH-wildtype (CNS WHO grade 4) (20/155; 13%), diffuse astrocytic glioma (CNS WHO grade 3), NOS, and diffuse hemispheric glioma, H3 G34-mutant (CNS WHO grade 4) (14/155; 9% each). In addition, three cases of infant-type hemispheric glioma and three cases of astrocytoma, IDH-mutant (CNS WHO grade 4) were included (3/155; 2% each) (Fig. 1b). A detailed list of all 155 centrally reviewed cases can be found in Table 1. 127 of all 155 cases (82%) were successfully examined by MMR-IHC for expression of MSH2, MSH6, MLH1, and PMS2 (Fig. 1a). In 28/155 (18%), MMR-IHC could not be performed due to insufficient tumor tissue amount or was inconclusive due to very low tumor cell density. 16 of these 28 patients (57%) had undergone stereotactic biopsy and were subsequently diagnosed with DMG.

**Table 1:** Patient characteristics of all centrally reviewed pediatric high-grade glioma cases screened for inclusion in the HIT-HGG-2013 trial (n = 155)

Baseline characteristics	n = 155	100%
Sex		
Male	92	59%
Female	61	39%
n.a	2	1%
Age at diagnosis		
Median	10.00 years	
Range	0.29–18 years	

Classification	n = 155	100%
Initially classified according to WHO 2016; reclassified	107	
Initially classified according to WHO 2021	48	

Diagnosis (WHO 2021)	n = 155	100%
Infant-type hemispheric glioma	3	2%
Astrocytoma, IDH-mutant (CNS WHO grade 3)	4	3%
Diffuse astrocytic glioma (CNS WHO grade 3), NOS	14	9%
Astrocytoma, IDH-mutant (CNS WHO grade 4)	3	2%
Diffuse pediatric-type high-grade glioma, H3-wildtype and IDH-wildtype (CNS WHO grade 4)	20	13%
Glioblastoma, IDH-wildtype (CNS WHO grade 4)	2	1%
Glioblastoma, IDH-wildtype (CNS WHO grade 4), subtype epithelioid glioblastoma	1	1%
Glioblastoma, IDH-wildtype (CNS WHO grade 4), subtype gliosarcoma	1	1%
Diffuse midline glioma, H3 K27-altered (CNS WHO grade 4)	82	53%
Diffuse hemispheric glioma, H3 G34-mutant (CNS WHO grade 4)	14	9%
Diffuse astrocytic glioma, H3-wildtype and IDH-wildtype (CNS WHO grade 4), NOS	5	3%
Diffuse astrocytic glioma, IDH-wildtype (CNS WHO grade 4), NOS	4	3%
Diffuse astrocytic glioma (CNS WHO grade 4), NOS	2	1%

Immunohistochemistry	n = 155	100%
Performed	127	82%
Not performed/inconclusive	28	18%

Information on Predisposition	n = 155	100%
Available	97	63%
Not available	58	37%

*IDH* isocitrate dehydrogenase, *NOS* not otherwise specified

### MMR protein loss in pedHGG

**Fig. 2:**
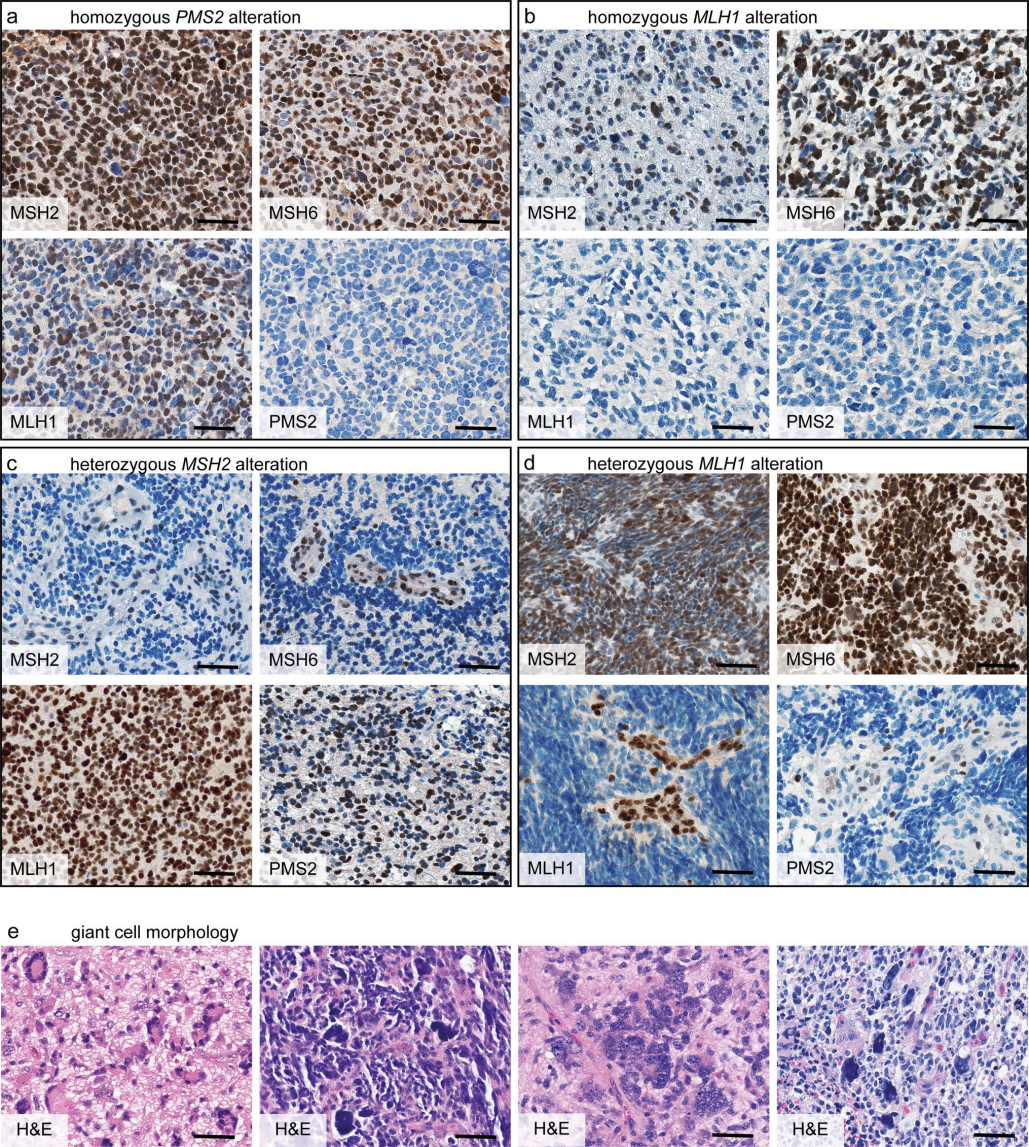
Immunohistochemical screening for mismatch repair deficiency in pediatric high-grade glioma. **a**–**d** Two control cases (**a**, **b**) and two HIT-HGG-2013 cases (**c**, **d**) with lost MMR protein expression are representatively displayed. **a**, **b** Both control cases are diagnosed with CMMRD. **a** Case CTRL 6 shows a complete loss of PMS2 expression, whereas the expression of MSH2, MSH6, and MLH1 is preserved. The underlying mechanism is a homozygous *PMS2* alteration. **b** In case CTRL 1, the expression of MSH2 and MSH6 is retained. MLH1 and PMS2 protein cannot be detected due to a homozygous *MLH1* alteration. **c** In case ID 90, a patient with confirmed LS, a heterozygous *MSH2* alteration causes a loss of expression of MSH2 and MSH6 in the tumor cells but not in the endothelial cells. MLH1 and PMS2 IHC are retained. **d** Patient ID 29 resembles CTRL 1 (in **b**) but here the loss of MLH1 and PMS2 is restricted to the tumor cells; endothelial cells are positive for all four MMR proteins. The underlying pathomechanism is a heterozygous *MLH1* alteration in the context of LS. **e** Variances in the appearance of giant cell morphology in four different H&E stained tissue samples. Case IDs in order from left to right: 26 (InDel in *MSH6*; CMMRD), 29 (SNV in *MLH1*, LS), 84 (InDel in *MSH2*; LS) and 137 (hypermethylation of *MSH2*; no germline affection). Cases are selected due to their different underlying genetic alterations and germline affections. Scale bars equate 50 µm. *CMMRD* constitutional mismatch repair deficiency, *CTRL* control, *MMRD* mismatch repair deficiency, *H&E* hematoxylin and eosin, *IHC* immunohistochemistry, *InDel* insertion/deletion, *LS* Lynch syndrome, *SNV* single-nucleotide variant

Ten of the 127 evaluable tumors (8%) showed loss of either one or two MMR proteins in tumor tissue defining MMRD and potentially indicating underlying germline MMR gene alterations (referred to as germline MMRD) (6/10; 60% loss of MSH2 and MSH6, 3/10; 30% loss of MLH1 and PMS2, 1/10; 10% loss of MSH6) (Fig. 1c; interpretation of IHC results in Fig. S2). Cases with homozygous pathogenic MMR gene alterations in the IHC control and screening group demonstrated a complete loss of expression in the tumor and surrounding brain tissue, while cases with heterozygous alterations showed retained MMR protein expression in the tumor vessels (Fig. 2a-d). 8/10 (80%) tumors revealed multinucleated giant cells (Fig. 2e), an unspecific feature commonly associated with MMRD in pedHGG [20].

6/10 (60%) patients were male; 4/10 (40%) female with a median age of 12.5 (range 6–17) years. Six of these patients (60%) were diagnosed with diffuse pediatric-type HGG, H3-wildtype and IDH-wildtype (CNS WHO grade 4), two with astrocytoma, IDH-mutant (CNS WHO grade 4) and two with diffuse hemispheric glioma, H3 G34-mutant (CNS WHO grade 4) (20% each) (Fig. 1b). Detailed MMRD patient characteristics are depicted in Table 2.

**Table 2:** Patient characteristics of all pedHGG patients with mismatch repair deficiency (MMRD) (n = 10)

Baseline characteristics	n = 10	100%
Sex		
Male	6	60%
Female	4	40%
Age at diagnosis (years)		
Median	12.5	
Range	6–17	

Classification	n = 10	100%
Initially classified according to WHO 2016; reclassified	4	40%
Initially classified according to WHO 2021	6	60%

Diagnosis (CNS WHO 2021)	n = 10	100%
Astrocytoma, IDH-mutant (CNS WHO grade 4)	2	20%
Diffuse pediatric-type high-grade glioma, H3-wildtype and IDH-wildtype (CNS WHO grade 4)	6	60%
Diffuse hemispheric glioma, H3 G34-mutant (CNS WHO grade 4)	2	20%

Immunohistochemistry	n = 10	100%
Status		
Performed	10	100%
Not performed/inconclusive	0	0%
Results		
(Homozygous) loss of MSH6	1	10%
(Heterozygous) loss of MSH2 and MSH6	6	60%
(Heterozygous) loss of MLH1and PMS2	3	30%

Information on genetic predisposition	n = 10	100%
Availability		
Provided	10	100%
Not provided	0	0%
Syndrome		
CMMRD	1	10%
Lynch	6	60%%
Li-Fraumeni	1	10%%
None	2	20%

Molecular genetic analysis	n = 10	100%
Frequently altered genes (in ≥ 3/10 cases)—MMR genes excluded		
*ATM*	3	30%
*ATRX*	8	80%
*CDKN2A*	3	30%
*NF1*	5	50%
*NOTCH1*	4	40%
*NOTCH2*	3	30%
*PDGFRA*	4	40%
*PIK3CA*	4	40%
*SETD2*	8	80%
*SPEN*	3	30%
*TP53*	10	100%
TMB (mut/Mb)	n = 10	100%
Median	17.8	
Range	7.20–254	
TMB > 10	9	90%
TMB < 10	1	10%
MSI	n = 9	
Median	10.4	
Range	4.50–25.41	
Percentage of unstable MS sites > 5	8	89%
Percentage of unstable MS sites > 10	5	56%
ALT phenotype	n = 10	100%
Yes	8	80%
No	2	20%

Tumor localization	n = 10	100%
Lobes		
Frontal	4	40%
Parietal	3	30%
Temporal	3	30%
Occipital	1	10%

Survival data (further details in Supplementary Table 3 and 4)	n = 10	100%
Vital status		
Alive	4	40%
Dead	6	60%
Relapse		
Yes	8	80%
No	2	20%

Therapy		
Initial	n = 10	100%
Temozolomide	9	90%
Lomustine	1	10%
Bevacizumab	1	10%
Radiotherapy	9	90%
Proton radiation	3	30%
After relapse	n = 8	100%
Nivolumab	6	75%
Ipilimumab	4	50%
Bevacizumab	3	38%
Lomustine	2	25%
Temozolomide	1	13%
Rapamycin	1	13%
Selumetinib	1	13%
Radiotherapy	2	25%
Proton radiation	1	13%

MMRD patients were identified by loss of mismatch repair protein expression in MMR-immunohistochemistry

*ALT* alternative lengthening of telomeres, *CMMRD* constitutional mismatch repair deficiency, *IDH* isocitrate dehydrogenase, *MS(I)* microsatellite (instability), *mut/Mb* mutations per megabase, *TMB* tumor mutational burden

### Molecular genetic analysis of MMR genes

**Fig. 3:**
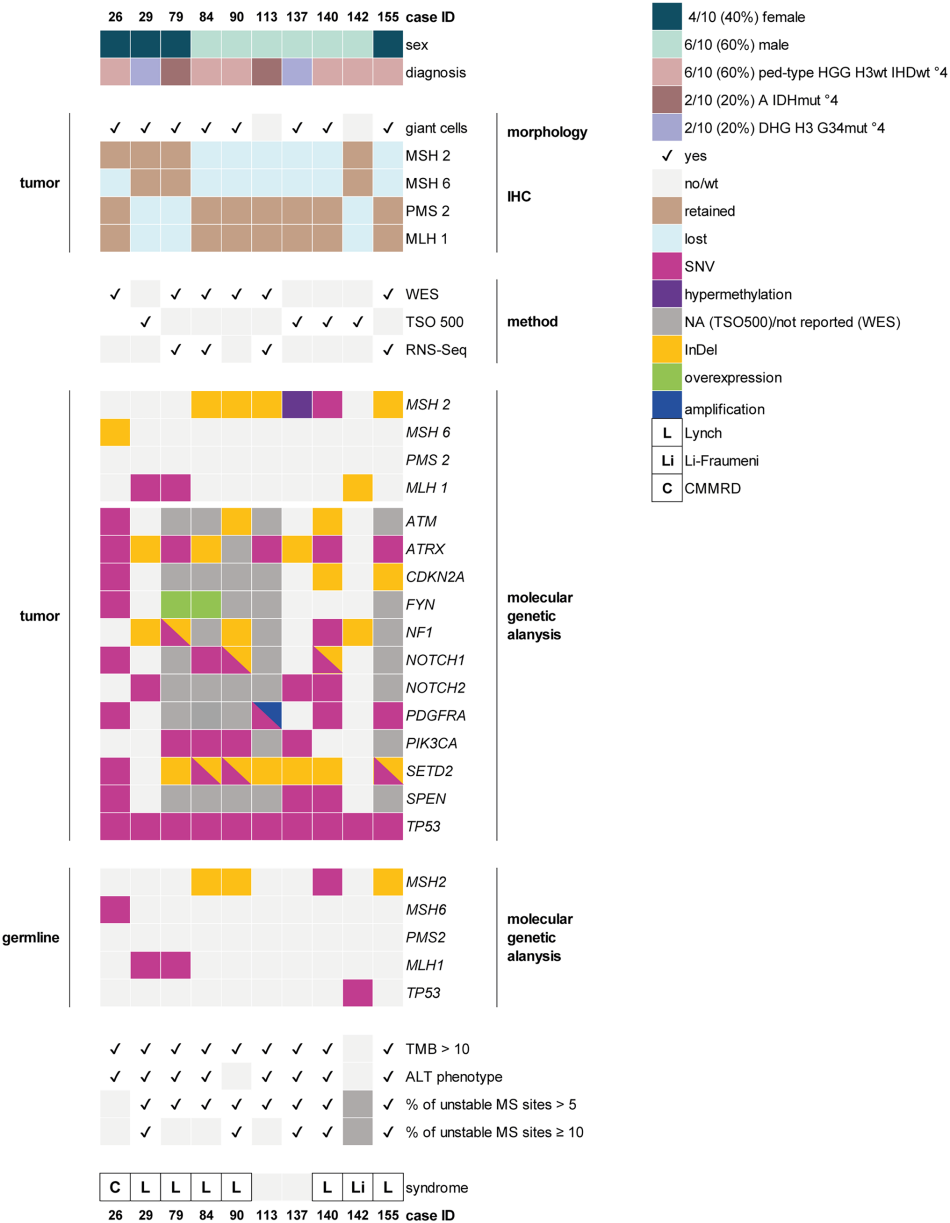
Oncoplot of cases with mismatch repair deficiency. A detailed case-related listing of clinical and histo-morphological characteristics and summary of most frequent molecular genetic findings. The latter includes results from tumor and germline analyses. Somatic alterations in genes other than *MSH2*, *MSH6*, *MLH1,* and *PMS2* are displayed when detected in three or more MMRD cases. *A IDHmut °4* astrocytoma, IDH-mutant (CNS WHO grade 4), *ALT* alternative lengthening of telomeres, *CMMRD* constitutional mismatch repair deficiency, *DHG H3 G34mut °4* diffuse hemispheric glioma, H3 G34-mutant (CNS WHO grade 4), *IDH* isocitrate dehydrogenase, *IHC* immunohistochemistry, *InDel* insertion/deletion, *MS* microsatellite, *NA* not analyzed, *ped-type HGG H3wt IDHwt °4* pediatric-type high-grade glioma, H3-wildtype and IDH-wildtype (CNS WHO grade 4), *Seq* sequencing, *SNV* single-nucleotide variant, *TMB* tumor mutational burden, *TSO 500* TruSight Oncology 500, *WES* whole exome sequencing, *wt* wildtype

**Fig. 4:**
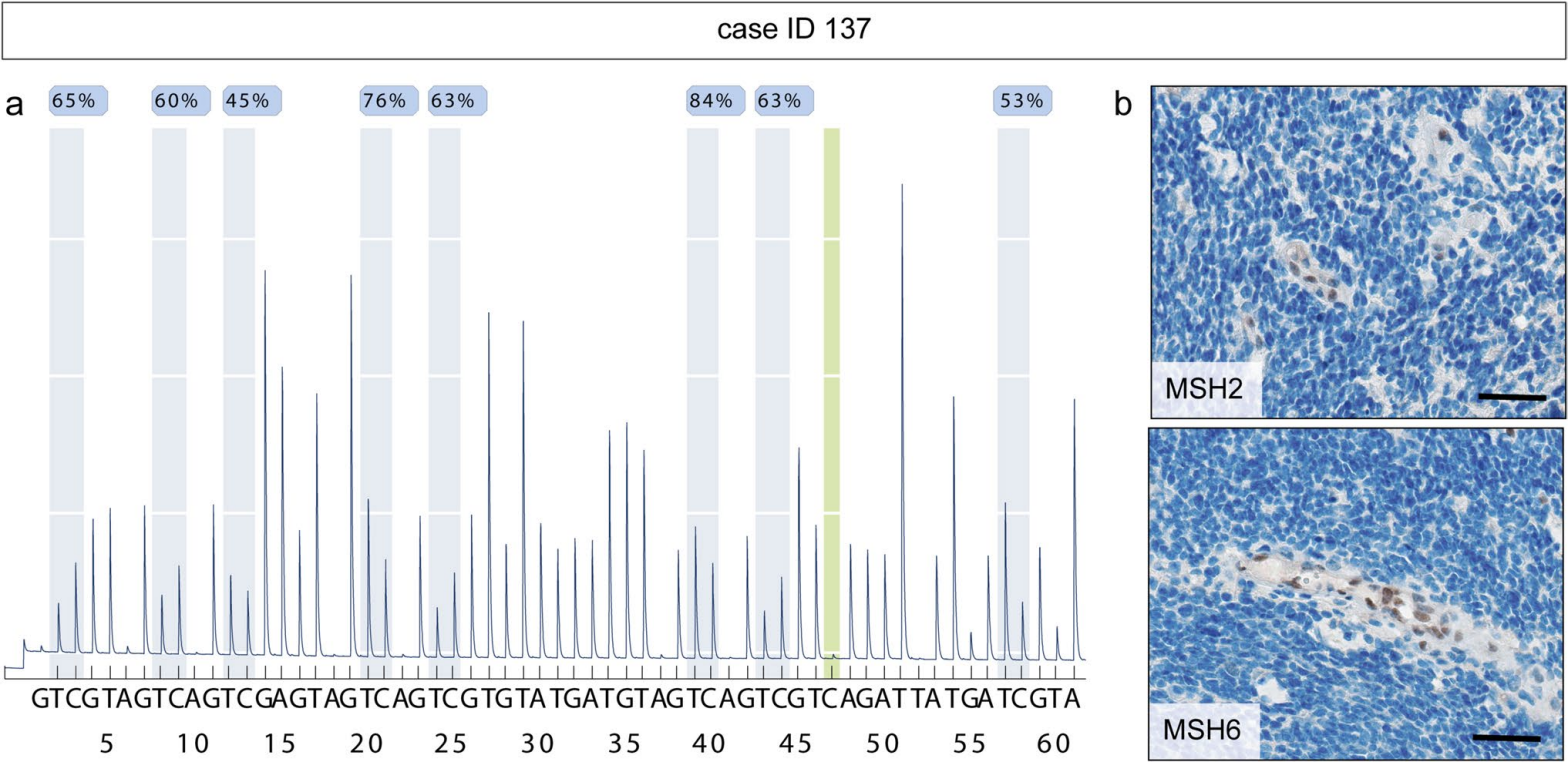
Hypermethylation of *MSH2* as alternative epigenetic silencing. **a** Diagram of *MSH2* gene pyrosequencing in tumor tissue of case ID 137. The first eight analyzed CpG dinucleotide positions are displayed. The average methylation of all eight positions was 63.6%. As bisulfite conversion control, the DNA nucleobase cytosine at position 47 is highlighted in green. **b** Immunohistochemistry reveals a loss of MSH2 and MSH6 protein expression in the tumor cells. Intratumoral vessels show preserved MSH2 and MSH6 protein expression

Alterations in corresponding MMR genes were detected by in-depth molecular genetic analyses of DNA extracted from tumor tissue (WES or TSO 500, Fig. 3). While three cases with combined immunohistochemical loss of MLH1 and PMS2 revealed *MLH1* gene alterations, one case with loss of MSH6 showed a *MSH6* alteration and six cases with combined loss of MSH2 and MSH6 were altered in *MSH2*. In all cases but one the alterations found were either single-nucleotide variants (SNV) (3/10, 30%) or insertions/deletions (InDel) (6/10, 60%). In one case (ID 137) with distinct combined MSH2 and MSH6 loss, no corresponding genetic alteration could be detected by DNA panel analysis. As silencing of *MLH1* by promoter methylation is known to cause loss of MLH1 protein expression in colorectal cancer [5, 19, 30], we designed a pyrosequencing assay to test for *MSH2* methylation as an alternative mechanism. Indeed, ID 137 showed hypermethylation of *MSH2* gene promotor with an average methylation of 63.6% (positive control: 94.1%, negative control: 2.3%) (Figs. 4 and S3).

Besides MMR gene alterations, analysis of tumor tissue of the ten MMRD cases revealed frequent genetic variants in *ATRX* (8/10, 80%), *NF1* (5/10, 50%), *PDGFRA* (4/10, 40%), *PIK3CA* (4/10, 40%), *SETD2* (8/10, 80%), and *TP53* (10/10, 100%). 9/10 MMRD cases (90%) showed an elevated tumor mutational burden (TMB) with values > 10 mutations per megabase (mut/Mb) (median 17.8 mut/Mb, range 7.20–254 mut/Mb).

8/9 cases with available data (98%) showed > 5% of unstable MS sites; 5/8 even > 10% (median 10.4, range 4.50–25.41). Alternative lengthening of telomeres (ALT-phenotype), representing an independent molecular characteristic associated with MMRD in pedHGG [22, 25, 39], here determined by the simultaneous detection of an *ATRX* and *TP53* gene alteration, was detected in 8/10 cases.

In all ten cases with MMR-IHC loss, germline testing was recommended by the treating physicians. In six patients (60%), subsequent genetic testing of leukocyte DNA confirmed the heterozygous pathogenic MMR variants in tumor tissue as germline variants, so that an underlining LS were diagnosed. In one case (10%), a homozygous *MSH6* variant could be confirmed as germline variant corresponding to CMMRD. One patient (10%) with MMRD was diagnosed with Li-Fraumeni-Syndrome but no LS or CMMRD. 2/10 patients including ID 137 (20%) had genetic alterations that were restricted to the tumor. A detailed report of al MMR gene associated variants can be found in Table S1.

### Screening test statistics

To evaluate the efficiency of MMR-IHC to screen for germline MMRD, MMR staining results were compared to results from germline CPS testing available in MMP2.0, INFORM, and HIT-HGG-2013 databases. Detailed statistical comparison is displayed in Table S2. In our cohort of 127 central-review confirmed pedHGG patients with evaluable MMR-IHC, staining of MSH2, MSH6, MLH1, and PMS2 successfully identified all present cases with LS and CMMRD resulting in a test sensitivity of 100% [95% confidence interval (CI) 0.6457 to 1.0000]. In addition to cases with germline affection, IHC also detected cases with exclusive somatic MMR gene alterations. These non-germline findings contributed to a germline MMRD screening sensitivity of 96% (95% CI 0.8875 to 0.9890) and a positive predictive value (PPV) of 70% (95% CI 0.3968 to 0.8922). None of the 71 cases with immunohistochemically retained MMR protein expression and available germline sequencing data had a MMRD syndrome, resulting in a negative predictive value of 100% (95% CI 0.9487 to 1.0000).

### Treatment regimes

9/10 patients were initially treated with radiotherapy and adjuvant temozolomide. Of eight patients with confirmed tumor recurrence, six patients received checkpoint inhibitors (either nivolumab only or in combination with ipilimumab) at relapse.

### Survival analyses

Median EFS and OS of the ten MMRD patients was 9.2 months (95% CI 5.8–12.7 months) and 14.1 months (CI 10.0–18.2 months), respectively (Table S3). In IDH-mutant astrocytomas, median EFS and OS of two patients with MMRD were reduced compared to six patients of the control cohort diagnosed with IDH-mutant astrocytomas lacking MMRD (EFS: 3.9 months vs. 23.1 months, CI (matched control) 16.7–29.5 months, p = 0.004; OS: 8.7 months vs. 31.0 months, CI 19.6–42.4 months; Table S3).

Since six patients with MMRD received checkpoint inhibitors only after progression of their pedHGG, EFS without checkpoint inhibition and PPS with checkpoint inhibition were compared with each other and with EFS and PPS of control patients (Table S4).

## Discussion

For the first time, we prospectively evaluated the use of IHC as a potential screening method for germline MMRD in pedHGG. In our cohort of 127 central-review confirmed pedHGG patients with evaluable MMR-IHC, staining of MSH2, MSH6, MLH1, and PMS2 successfully identified all present cases with LS and CMMRD. In addition to cases with germline affection, IHC also detected cases with exclusive somatic MMR gene alterations. Of note, these patients may also qualify for treatment adaptation toward checkpoint inhibition instead of cytotoxic therapy, as it is frequently beneficial for pedHGG patients with germline MMRD [4, 9, 36]. Indeed, 2/3 cases with exclusive somatic MMR alterations showed a molecular signature characteristic for MMRD with elevated TMB, MSI, and ALT phenotype [39] indicating the use of checkpoint inhibition [4, 9, 10, 36, 42]. In our series, IHC detecting protein expression independently of the underlying pathomechanism appeared even superior to identify MMRD compared to extensive molecular panel diagnostics.

Previously, in a study by Chung et al., the diagnostic ability to detect MMRD was compared between Low-pass Genomic Instability Characterization (LOGIC) assay, TMB analysis, traditional MSI panel testing, and IHC. This comparison was performed in 56 mixed childhood cancer cases including 27 CNS tumors [6]. In this mixed cancer cohort, the LOGIC assay reached the highest sensitivity to detect MMRD (100%). However, the sensitivity of IHC (86%) was superior to traditional MSI panel testing (14%) and TMB analysis (80%). With focus on CNS tumors, IHC reached a sensitivity of 93%; 25/27 MMRD cases were successfully identified by IHC. Interestingly, IHC could be successfully performed in eight cases with insufficient sequencing coverage during low-pass whole-genome sequencing. Since in our study, MMR-IHC detected MMRD with 100% sensitivity in 127 investigated cases, the performance of the different assays may vary depending on the respective type of cancer analyzed.

**Fig. 5:**
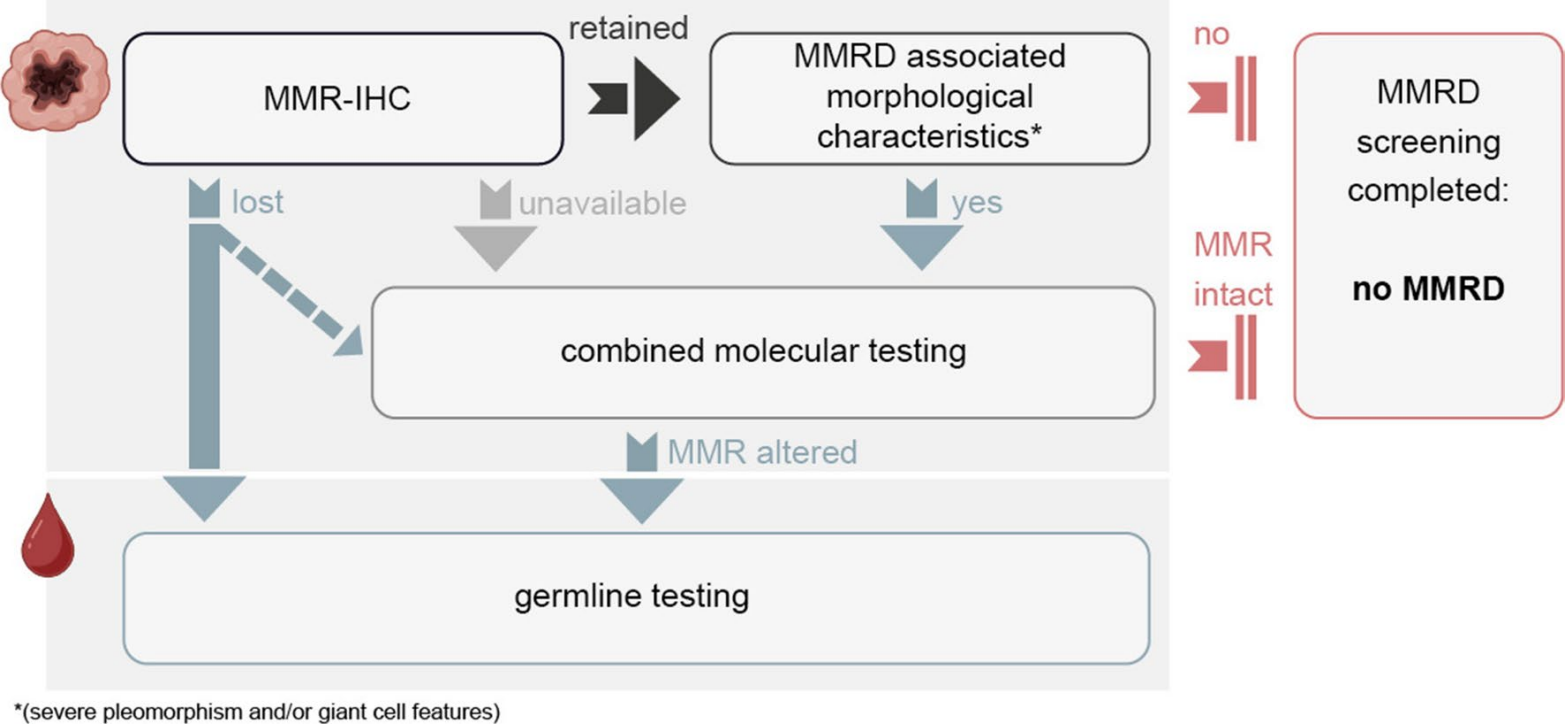
Adapted diagnostic workflow for routine MMRD screening in pedHGG. We would like to recommend performing MMR-IHC in all cases of pedHGG. In cases with unavailable MMR-IHC, or MMRD-associated tumor cell morphology (severe pleomorphism and/or giant cell features), molecular testing should be performed—if feasible—to identify any underlying MMR gene alteration with the need for subsequent MMR gene germline testing. Based on the present findings, MMR gene germline testing may be promptly initiated in cases of MMR-IHC loss; however, prior molecular analysis of tumor tissue for MMR gene alterations can be performed at the investigator’s discretion. Ideally, a combination of molecular assays covering all potential types of alterations including hypermethylation is implemented. Germline testing is recommended for all MMRD patients. *MMR(D)* mismatch repair (deficiency), *IHC* immunohistochemistry

Loss-of-function alterations within the MMR genes can lead to the expression of a defective protein. These cases appear to be rare. Hechtman et al. examined this phenomenon in a cohort predominantly comprising cases of colorectal carcinoma and uterine endometrioid carcinoma. Their study reported that approximately 6% of MSI-high (MSI-H) cases exhibited retained MMR protein expression [18]. Similarly, Chen et al. observed that 7% of MMR-deficient colorectal carcinoma cases (6 out of 82) showed intact MMR immunohistochemical staining [7]. In our study, in all 71 cases with retained protein expression, DNA sequencing analysis never identified any alterations in the MMR genes. However, as MMRD is predominantly studied in tumors other than pedHGG [7, 18], evidence regarding the frequency of this phenomenon in pedHGG remains limited. To mitigate the risk of missing rare MMRD cases with retained MMR protein expression, we recommend incorporating the evaluation of MMRD-associated morphological characteristics [20] into the algorithm of pedHGG diagnostics (Fig. 5).

The WHO 2021 recommends immunohistochemical MMRD testing in pedHGG cases with severe pleomorphism or giant cell features [20]. Based on our findings, we suggest an adapted diagnostic workflow for MMRD screening in pedHGG (Fig. 5). Instead of performing MMR-IHC only in pedHGG cases with MMRD-associated morphological characteristics, we suggest performing MMR-IHC in all pedHGG cases at first-line, as in our study, 2/10 MMRD cases lacked MMRD-associated morphological features. In cases with retained MMR protein expression, the mentioned morphological characteristics can then be used to identify rare cases with loss-of-function alterations by further molecular investigations.

The LOGIC assay using low-pass whole-genome sequencing to detect microsatellite instability with much greater sensitivity than traditional MSI panels in childhood chancers seems to represent a robust tool to investigate pedHGG cases with unavailable or difficult to interpret IHC [6]. Traditional MSI panels were originally developed for colorectal cancer [43], and their gene selection and cut-off values may not be directly applicable to other cancer types, particularly pediatric malignancies. A study by Hause et al. highlights the presence of instability signatures and cancer-specific properties of MSI [17]. Furthermore, elevated MSI levels may occur via independent mechanisms [32]. In cases with immunohistochemical MMR protein loss, either WES or TSO 500 determined the percentage of unstable MS sites retrospectively. While in colorectal, esophageal, and lung cancer, MSI-H is defined as > 10% unstable microsatellite sites [33], the present study provides the first evidence that the cut-off for MSI-H might require to be set significantly lower (> 5%) for pedHGG.

We are the first to describe *MSH2* hypermethylation as an alternative epigenetic silencing of MSH2 and MSH6 expression in pedHGG. This alternative mechanism had not been determined by routine molecular diagnostics [40]. However, since *MSH2* hypermethylation is reported to occur in 24% of MSH2-deficient colorectal carcinoma [29], testing for *MSH2* gene promotor hypermethylation should always be performed when a loss of MSH2 and MSH6 protein expression is found by IHC, but no corresponding underlying genetic variant is detected by DNA panel sequencing.

Several molecular methods are available to investigate the underlying MMR gene alterations, including NGS, WES/WGS, copy-number variation (CNV) analysis, and pyrosequencing. However, no single assay can comprehensively detect all types of alterations. CNV analysis is suited for detecting amplifications and deletions, while NGS and WES/WGS are more adept at identifying point mutations, small insertions, and deletions. Pyrosequencing, on the other hand, is highly effective for detecting gene promoter hypermethylation. Therefore, to achieve comprehensive coverage of genetic mechanisms, a combination of molecular assays is necessary. For instance, NGS coupled with pyrosequencing could provide a robust approach. This multi-faceted approach underscores the importance of elaborating diagnostic workflows to maximize sensitivity and to ensure that no underlying genetic mechanism is overlooked (Fig. 5).

Despite the high PPV of the here evaluated screening method, in routine care, MMRD detection in the tumor does not necessarily result in germline testing and counseling of the family [32]. In case of pediatric oncological patients, the barriers for further germline testing might be clearly lower. All patients with MMRD detected in MMR-IHC in this present study underwent germline testing and counseling. This is especially important, since in four of seven patients with germline involvement, MMR-IHC results and subsequent germline testing led to the initial diagnosis of LS in the affected families.

Germline testing of the patients with loss of MMR-IHC identified a LS or CMMRD in 70% of MMRD cases. In these patients’ families, the diagnosis of a CPS enables predictive testing of all relatives at risk. Subsequently, intensive, life-saving surveillance programs can be offered to all variant carriers in the families to prevent cancer or to identify early stages with good prognosis [11, 16, 42].

Our sequencing revealed *MSH2* as the most commonly altered gene which is in accordance with the reported findings by the IRRDC [12]. The present observation of the frequently co-altered genes *ARTX, NF1,* and *TP53* is also consistent with the previous findings in CNS tumors with underlying CMMRD [12, 42].

Other than any molecular analysis, MMR-IHC can be easily, rapidly, and cost-effectively performed in neuropathology institutes world-wide [2, 3, 42]. In the present pedHGG series, MMR-IHC screening reduced the number of the cases in which testing for germline MMRD was required from 127 to 10 patients, i.e., to approximately 8%. Considering the 28 patients with non-performable/-evaluable MMR-IHC, the rate of recommended germline testing would still be only 25% of pedHGG patients.

However, technical limitations of routine IHC screening for MMRD in pedHGG patients must be taken into account. If the sample is composed predominantly of resident MMR protein-positive non-neoplastic glial cells or dense immune cell infiltration, loss of MMR protein expression in small pedHGG samples may be missed. Thus, personal experience and caution is required to select cases for germline testing. However, this also applies to the interpretation of results of molecular analyses, where quality may also be limited by low tumor cell count. Consequently, in cases with only very small tissue samples and/or low tumor cell density, germline testing should be recommended at a low threshold. This applies especially when they are diagnosed with IDH-mutant astrocytoma [41] or diffuse pediatric-type HGG, H3-wildtype and IDH-wildtype [40].

Difficult-to-reach tumors located in midline are frequently associated with very small biopsy samples. As none of the screened DMG in this cohort showed a loss of MMR-IHC, it may be assumed that there is no association between DMG and MMRD. This observation needs to be verified in larger patient cohorts but previous findings reporting predominant MMRD association with diffuse pediatric-type HGG, H3-wildtype and IDH-wildtype and IDH-mutant astrocytoma strongly support the absence of MMRD in DMG patients [40]. Furthermore, individual cases of H3 G34-mutant pedHGG with germline MMRD as found in the present series (ID 29) had been reported previously [26].

We additionally assessed potential survival differences between MMRD-associated pedHGG and pedHGG controls lacking MMRD. Despite small patient numbers, EFS and OS of IDH-mutant astrocytoma with loss of MMR-IHC were inferior compared to IDH-mutant astrocytoma controls without underlying MMRD confirming previously reported results [41].

Combined immunotherapy with nivolumab and ipilimumab provides a promising treatment option for patients with MMRD/MSI-H metastatic colorectal cancer [31]. There is evidence for a benefit from immune-directed/synergistic salvage therapies in Replication-Repair-Deficient HGG [9]. The present data may support a potential benefit of checkpoint inhibition in pedHGG MMRD patients although the patient numbers are very small, and patients received checkpoint inhibition only as second-line treatment approach. IDH inhibitors like ivosidenib and the particularly blood–brain barrier permeable vorasidenib may be a suitable combination partner for checkpoint inhibition to improve survival of MMRD patients with IDH-mutant astrocytoma [27].

As MMR-IHC demonstrated a sensitivity of 100% in detecting MMRD, germline genetic testing can be promptly initiated for pedHGG patients with loss of MMR protein expression (Fig. 5), as well as for their relatives at-risk, within 1–2 days. This rapid turnaround facilitates early identification of germline cancer predisposition syndromes and may influence clinical management. Our study may indicate that pedHGG MMRD patients may indeed benefit from checkpoint inhibitor therapy. However, further clinical studies are required to evaluate the efficacy of checkpoint inhibitor therapy as a first-line treatment in pedHGG patients. These future studies could potentially lead to the recommendation of checkpoint inhibitor therapy as a standard first-line option for all pedHGG patients with confirmed MMRD at diagnosis.

In conclusion, IHC represents an easy to perform, cost-effective and fast method to screen for germline MMRD in pedHGG with global applicability and both high sensitivity and specificity. We therefore recommend incorporating MMR-IHC into routine diagnostics of pedHGG. Positive results necessitate patient information about potential underlining CPS and, if desired, individual genetic testing and family counseling to offer life-saving surveillance measures to all germline variant carriers in the families. Furthermore, identified patients may benefit from checkpoint inhibition therapy even at first line.

## Supplementary Information

Below is the link to the electronic supplementary material.

Supplementary file1 (XLSX 21 KB)

Supplementary file2 (PDF 1921 KB)

## Data Availability

Requests for specific analyses or data, including access to de-identified individual participant information collected during the study, will be considered by L.L.F., G.H.G., and C.M.K. from 3 months after publication of the manuscript. To gain access, researchers need to provide a reasonable, methodologically sound proposal and to sign a data access agreement. Proposals should be directed to the corresponding author (lea.friker@ukbonn.de).
