# Supplementary material for: MSH2, MSH6, MLH1, and PMS2 immunohistochemistry as highly sensitive screening method for DNA mismatch repair deficiency syndromes in pediatric high-grade glioma
Source: Acta Neuropathol. 2025 Feb 2;149(1):11. doi: 10.1007/s00401-025-02846-x (PMC11788232; doi:10.1007/s00401-025-02846-x)
Supplement: Supplementary file 2 — Supplementary file2 (PDF 1921 KB) [file 401_2025_2846_MOESM2_ESM.pdf]

Supplementary Figure 1

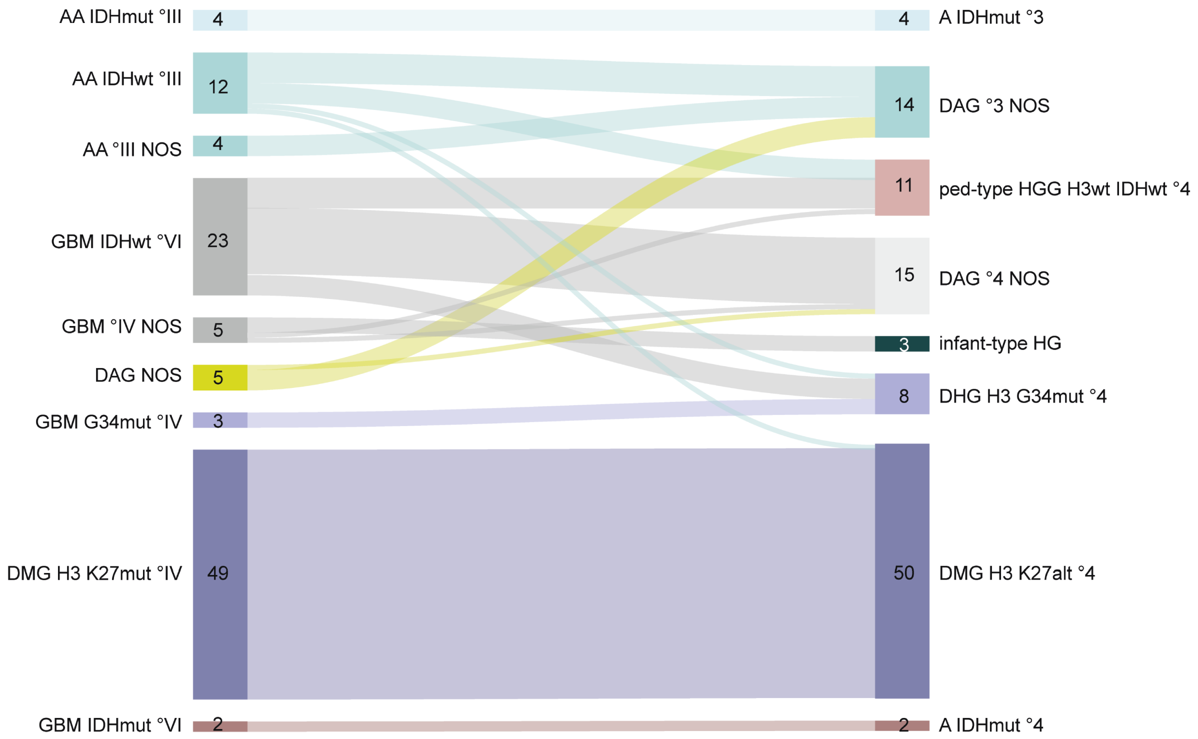

Fig. S1 Shift of diagnoses between WHO classification of CNS tumors 2016 and 2021. 107 cases were initially diagnosed according to the classification of 2016 (left-hand side) and later reclassified according to the currently valid classification of 2021 (right-hand side). The shift of diagnoses is displayed in a sankey diagram. The plot was created using SankeyMATIC.com

Abbreviations: AA °III = anaplastic astrocytoma (WHO grade III); A IDHmut °3/°4 = astrocytoma, IDH-mutant (CNS WHO grade 3/4); DAG = diffuse astrocytic glioma; DHG H3 G34mut °4 = diffuse hemispheric glioma, H3 G34-mutant (CNS WHO grade 4); DMG H3 K27alt °4 = diffuse midline glioma, H3 K27-altered (CNS WHO grade 4); DMG H3 K27mut °IV = diffuse midline glioma, H3 K27-mutant (WHO grade IV); GBM = glioblastoma; GBM G34mut °IV = glioblastoma with G34 mutation (WHO grade IV); IDHmut/wt = isocitrate dehydrogenase-mutant /-wildtype; infant-type HG = infant-type hemispheric glioma; NOS = not otherwise specified; ped-type HGG H3wt IDHwt °4 = pediatric-type high-grade glioma, H3-wildtype and IDH-wildtype (CNS WHO grade 4)

Supplementary Figure 2

		immunhistochemistry			
		MSH2	MSH6	MLH1	PMS2
genetic alteration	MSH 2	-	-	+	+
	MSH6	+	-	+	+
	MLH1	+	+	-	-
	PMS2	+	+	+	-

Fig. S2 Interpretation of immunohistochemistry of MSH2, MSH6, MLH1, and PMS2. The observed protein expression losses of one or two mismatch repair proteins in immunohistochemistry directly indicate the altered gene

Supplementary Figure 3

a positive control

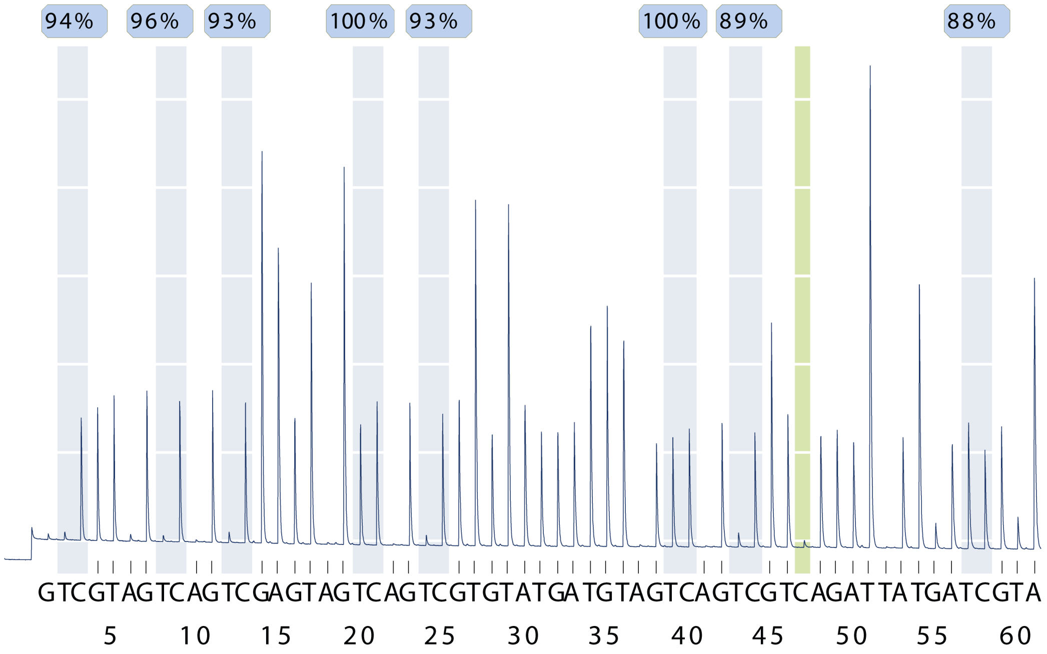

b negative control

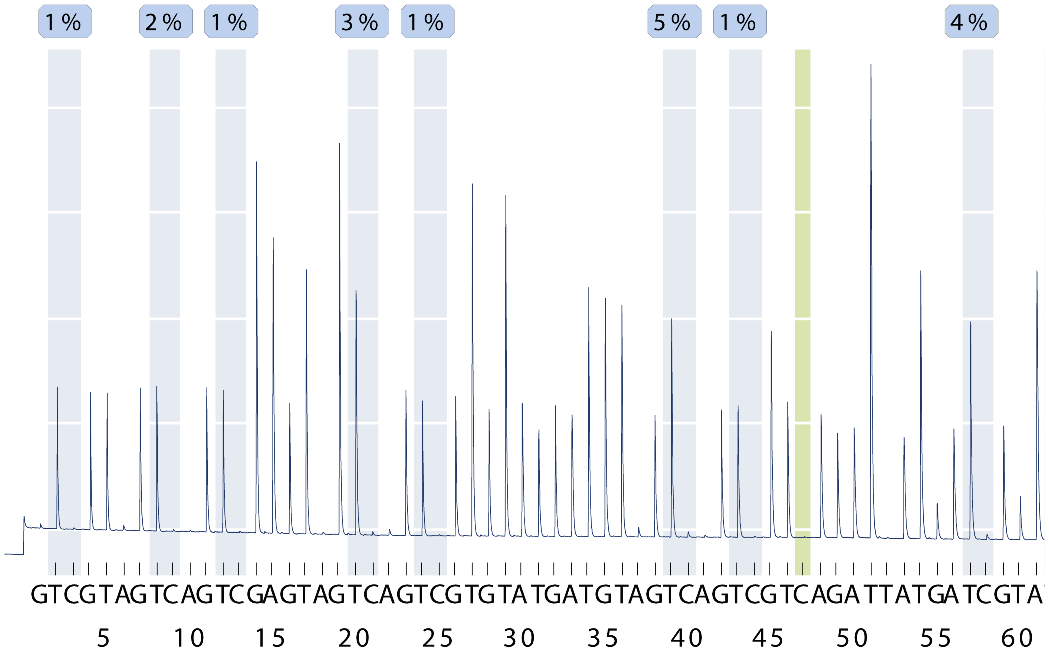

Fig. S3 DNA controls for *MSH2* pyrosequencing. (a) Diagram of *MSH2* gene pyrosequencing using bisulfite concerted ubiquitously methylated DNA as positive control and (b) bisulfite concerted *MSH2* unmethylated DNA from a control patient as negative control for *MSH2* gene hypermethylation. The first eight analyzed CpG dinucleotide positions are displayed. The average methylation of all eight positions was 94.1% for the positive control and 2.3% for the negative control. As bisulfite conversion control, the DNA nucleobase cytosine at position 47 is highlighted in green

Supplementary Table 1 – *provided separately*

Table S1 Detailed report of genetic drivers identified through next-generation sequencing of all mismatch repair deficient cases. The ClinVar platform was used to determine the variant significance. Only likely pathogenic or pathogenic alterations are reported.

Abbreviations: (CNN-)LOH = (copy number neutral) loss of heterozygosity; hm = hypermethylation; InDel = insertion/deletion; SNV = single nucleotide variant

Supplementary Table 2

		MMR-IHC	
		Loss of MMR protein expression	Preservation of MMR protein expression
**Genetic testing for MMRD (CPS data bases)**	MMRD syndrome	7	0
	No MMRD syndrome	3	71

Table S2 Screening test statistics. Parameters used to determine sensitivity and specificity of MMR-IHC as a screening method to detect MMRD in pedHGG in comparison to results from DNA sequencing provided by study databases

Abbreviations: CPS = cancer predisposition syndrome; IHC = immunohistochemistry; MMR(D) = mismatch repair (deficiency)

Supplementary Table 3

Cohorts studied	Median EFS (95% CI)	p value	Median OS (95% CI)	p value
PedHGG with IHC MMR protein loss (n=10)	9.2 mo (5.8 mo/12.7 mo)	0.393	14.1 mo (10.0 mo/18.2 mo)	0.514
PedHGG without MMRD (n=61)	11.7 mo (8.4 mo/15.1 mo)		17.4 mo (12.9 mo/21.9 mo)	
PedHGG with germline MMRD (LS/CMMRD) (n=7)	6.9 mo (6.8 mo/7.1 mo)	0.645	16.6 mo (10.3 mo/22.9 mo)	0.884
PedHGG without MMRD (n=61)	11.7 mo (8.4 mo/15.1 mo)		17.4 mo (12.9 mo/21.9 mo)	
PedHGG with Lynch (n=6)	6.9 mo (3.2 mo/10.5 mo)	0.528	14.1 mo (6.4 mo/21.8 mo)	0.852
PedHGG without MMRD (n=61)	11.7 mo (8.4 mo/15.1 mo)		17.4 mo (12.9 mo/21.9 mo)	
Diffuse pediatric-type HGG, H3/IDH-wildtype (CNS WHO grade 4) with germline MMRD (LS/CMMRD) (n=5)	6.9 mo (6.8 mo/7.1 mo)	0.758	16.6 mo (11.3 mo/21.9 mo)	0.690
Diffuse pediatric-type HGG, H3/IDH-wildtype (CNS WHO grade 4) without MMRD (n=35)	10.3 mo (8.2 mo/12.4 mo)		15.9 mo (7.2 mo/24.6 mo)	
Astrocytoma, IDH-mutant (CNS WHO grade 4) with IHC MMR protein loss (n=2)	3.9 mo	0.004	8.7 mo	0.004
Astrocytoma, IDH-mutant (CNS WHO grade 4) without MMRD (n=6)	23.1 mo (16.7/29.5 mo)		31.0 mo (19.6/42.4 mo)	

Table S3 Survival analysis of pediatric high-grade glioma patients with mismatch repair deficiency (MMRD) compared to a control cohort from the HIT-HGG-2007/-2013 trial lacking MMRD. In the analysis, we distinguish between MMRD, which is defined by the loss of MMR protein expression in immunohistochemistry, and MMRD that is also manifest in the germline as Lynch syndrome or CMMRD

Abbreviations: CI = confidence interval; IDH = isocitrate dehydrogenase; IHC = immunohistochemistry; (C)MMR(D) = (Constitutional) Mismatch Repair (Deficiency); LS = Lynch syndrome; pedHGG = pediatric high-grade glioma

Supplementary Table 4

Cohorts studied	Median EFS (95% CI)	p value	Median PPS (95% CI)	p value	Median OS (95% CI)	p value
PedHGG with MMRD and checkpoint inhibition (n=6)	6.9 mo (3.2 mo/10.5 mo)	0.246	9.7 mo (1.3 mo/ 18.1 mo)	0.513	14.1 mo (6.4 mo/21.9 mo)	0.615
PedHGG without MMRD (n=61)	11.7 mo (8.4 mo/15.1 mo)		5.9 mo (3.7 mo/8.0 mo)		17.4 mo (12.9 mo/21.9 mo)	
Diffuse pediatric-type HGG, H3/IDH-wildtype (CNS WHO grade 4) with MMRD and checkpoint inhibition (n=4)	6.9 mo (1.1 mo/12.7 mo)	0.237	9.7 mo (1.3 mo/ 18.1 mo)	0.805	14.1 mo (7.8 mo/20.4 mo)	0.396
Diffuse pediatric-type HGG, H3/IDH-wildtype (CNS WHO grade 4) without MMRD (n=35)	10.3 mo (8.2 mo/12.4 mo)		5.0 mo (1.6 mo/8.4 mo)		15.9 mo (7.2 mo/24.6 mo)	

Table S4 Survival of pediatric high-grade glioma patients with mismatch repair deficiency compared to a control cohort from HIT-HGG-2007/-2013 trial without cancer predisposition taking into account checkpoint inhibitor therapy

Abbreviations: CI = confidence interval; IDH = isocitrate dehydrogenase; MMRD = mismatch repair deficiency; pedHGG = pediatric high-grade glioma
